# Real-World Analysis of HRD Assay Variability in High-Grade Serous Ovarian Cancer: Impacts of BRCA1/2 Mutation Subtypes on HRD Assessment

**DOI:** 10.3390/biom15050745

**Published:** 2025-05-21

**Authors:** Giovanni Luca Scaglione, Valentina Lombardo, Maurizio Polano, Giuseppa Scandurra, Angela Pettinato, Corrado Giunta, Rosario Iemmolo, Paolo Scollo, Ettore D. Capoluongo

**Affiliations:** 1 Bioinformatics Unit, Istituto Dermopatico dell’Immacolata, IDI-IRCCS, 00167 Rome, Italy; g.scaglione@idi.it; 2 Department of Medical Oncology, A.O.E. Cannizzaro, 95126 Catania, Italy; lombardovalentina89@gmail.com (V.L.); giuseppa.scandurra@unikore.it (G.S.); 3 Faculty of Medicine and Surgery, “Kore” University, 94100 Enna, Italy; paolo.scollo@unikore.it; 4 Experimental and Clinical Pharmacology Unit, Centro di Riferimento Oncologico di Aviano, Istituto di Ricovero e Cura a Carattere Scientifico, 33081 Aviano, Italy; mpolano@cro.it; 5 Department of Pathological Anatomy, A.O.E. Cannizzaro, 95126 Catania, Italy; apettinato20@gmail.com; 6 Laboratory of Genomics, L.C. Laboratori Campisi, 96012 Avola, Italy; giuntacorrado@libero.it (C.G.); iemmolo.rosario@gmail.com (R.I.); 7 Department of Obstetrics and Gynecology, A.O.E. Cannizzaro, 95126 Catania, Italy; 8 Department of Molecular Medicine and Medical Biotechnology, Federico II University, 80131 Naples, Italy; 9 Department of Clinical Pathology, San Giovanni Addolorata Hospital, 00184 Rome, Italy

**Keywords:** BRCA, HRD, ovarian cancer

## Abstract

The HRD (Homologous Recombination Deficiency) test is considered a genomic alteration useful for guiding therapeutic decisions in patients with ovarian cancer. Some commercial and in house alternative “academic” tests are available. Recent findings indicate that not all *BRCA1/2* mutations determine the magnitude of HRD and that some patients carrying *BRCA1/2* mutations may exhibit indeterminate or even negative HRD scores. Furthermore, certain therapies (e.g., olaparib and bevacizumab) offer particularly pronounced benefits for high-grade serous ovarian cancer (HGSOC) patients harboring mutations in the DNA-binding domain (DBD) of *BRCA1/2*. The aim of the present study is to investigate the relationship between the HRD scores and *BRCA1/2* status of 51 HGSOC patients (50 *BRCA1/2* mutated and 1 wild type). The HRD status was assessed by means of shallow whole-genome sequencing and *BRCA1/2* status by the NGS pipeline. We did not find a correlation between the HRD status and type of *BRCA1/2* alterations. A strong correlation between the HRD score and age was found. Our paper underlines the need to introduce other biological factors within the algorithms of the HRD evaluation in order to better tailor the HRD status, harmonize the metrics of the HRD assessment, and personalize therapies.

## 1. Introduction

*BRCA1* and *BRCA2* are key tumor suppressor genes within the homologous recombination (HR) DNA repair pathway and represent the principal genetic determinants of hereditary breast and ovarian cancer (HBOC) syndrome.

Due to their significant clinical relevance, current ESMO-ESGO guidelines recommend comprehensive *BRCA1/2* and homologous recombination repair (HRR) testing at the time of the initial diagnosis. Pathogenic variants in *BRCA1/2* can occur across the entire coding sequence and encompass a wide range of mutation types, including nonsense, frameshift, and splice-site alterations, as well as large genomic rearrangements—such as deletions and duplications—and selected missense mutations that result in a loss of protein function [1].

While *BRCA1* is a protein that links the DNA damage response and DNA repair, *BRCA2* is essential in homologous recombination by mediating the recruitment of RAD51 recombinase to Double Strand Breaks (DSBs). Extensive characterization has clarified the domain structures of the *BRCA1* and *BRCA2* genes.

*BRCA1* has a highly conserved N-terminal Really Interesting New Gene (RING) domain with E3 ubiquitin ligase activity critical for *BRCA1*-BARD1 (*BRCA1*-Associated RING Domain protein 1) heterodimerization, a DNA-binding domain (DBD) that functions in DNA damage sensing and repair facilitation, and a C-terminal domain (BRCT) that binds phosphorylated proteins and mediates DNA end resection and G2/M checkpoint activation.

*BRCA2* has a central RAD51-binding domain (RAD51-BD) composed of eight BRC repeats serving as the primary interaction sites for RAD51 monomers, allowing filament formation on single-strand DNA (ssDNA); an additional RAD51 interaction site (TR2) that associates with RAD51 filaments; and a conserved C-terminal DBD mediating the *BRCA2* interaction with both ssDNA and double-stranded DNA (dsDNA) [2,3].

Recent studies have shown that different types of *BRCA1/2* mutations can yield distinct clinical outcomes, including variable responses to therapy. For instance, germline mutations in exon 11 of both genes are linked to a higher risk of ovarian over breast cancer, while the mutations in the DBD may confer greater benefit from specific treatments [3].

Although *BRCA1/2* mutations are the most extensively characterized genetic alterations associated with HR dysfunction and sensitivity to PARPis, other genes involved in DNA repair also contribute to the Homologous Recombination Deficient (HRD) status when altered.

Our sWGS pipeline represents a significant advancement in precision oncology by providing a nuanced understanding of the HRD status. It enables the identification of intermediate phenotypes (HR-Mild), which may have been overlooked in traditional assays. Moreover, the highest correlation with functional HRD testing (RAD51 foci) was found mainly in samples classified as HR-deficient [4], highlighting that a grey zone still exists in the metrics of HRD scores and that some unknown variables can affect all the assays. A better assessment of HRD has the potential to tailor therapeutic strategies, ensuring that patients receive treatments aligned with their unique genomic profiles.

The present report aims to show the evaluation of the relationship between the type of *BRCA1/2* mutation and the HRD status. In addition, to obtain the best personalized approach in determining the individual HRD score, individual clinical variables were also included in the evaluation model of HRD. Our results have therefore investigated the biological implications of the patient’s characteristics and *BRCA* mutational status on the genomic instability score.

## 2. Materials and Methods

### 2.1. Study Design and Patient Selection

Under the protocol approved by the Ethical Committee of Enna “Kore” University (prot. N 2573/2024, approved date: 8 February 2024), 51 patients were selected from a consecutive series of 58 patients diagnosed with ovarian cancer (OC) and undergoing surgery at the Department of Gynecology and Obstetrics of Cannizzaro Hospital (Catania) and were retrospectively enrolled. All patients provided informed consent allowing the use of their anonymized data for research purposes prior to enrollment. Clinical and demographic data were retrieved from the electronic medical records. Ovarian cancer tissue specimens from patients with an established *BRCA* mutational status [5] were subsequently assessed for the HR status using a previously validated shallow whole-genome sequencing (sWGS) pipeline [6].

### 2.2. DNA Isolation and Shallow Whole-Genome Sequencing (sWGS)

Following our previously validated protocol [6], genomic DNA was extracted from formalin-fixed paraffin-embedded (FFPE) tissues using the QIAamp DNA FFPE Advanced kit (QIAGEN, Hilden, Germany) according to the manufacturer’s instructions. DNA purity was assessed with a NanoDrop 1000 spectrophotometer (ThermoFisher Scientific, Waltham, MA, USA), and the DNA concentration was determined using the Qubit 1X dsDNA High Sensitivity (HS) Assay Kit on the Qubit^®^ Fluorometer 4.0 (Invitrogen Co., Life Sciences, San Francisco, CA, USA).

Library preparation for shallow WGS was performed using the Watchmaker DNA Library Prep Kit (Watchmaker Genomics, Boulder, CO, USA) in accordance with the manufacturer’s protocol.

Briefly, 100 ng of total DNA was enzymatically fragmented at 37 °C for 20 min to produce fragments of approximately 200 bp in size. End-repair and A-tailing were performed in the same incubation. Next, 15 µM xGen UDI-UMI Adapters (IDT, Coralville, IA, USA) were added to DNA fragments and incubated at 20 °C for 15 min in the adapter ligation step. Following a bead-based cleanup, library amplification was conducted as follows: initial denaturation at 98 °C for 45 s; 7 cycles of denaturation at 98 °C for 15 s, annealing at 60 °C for 30 s, and extension at 72 °C for 30 s; and a final extension at 72 °C for 60 s. The reaction was then held at 12 °C.

Library quality and integrity were evaluated using an Agilent D1000 ScreenTape for TapeStation system (Agilent Technologies, Santa Clara, CA, USA). The library concentration was measured using the Qubit 1X dsDNA High Sensitivity (HS) Assay Kit on the Qubit^®^ Fluorometer 4.0 (Invitrogen Co., Life Sciences, CA, USA), and the resulting values were used to calculate nanomolar concentrations.

Up to 40 samples were multiplexed and sequenced as paired-end reads on an Illumina NextSeq550 Dx System (Illumina, San Diego, CA, USA) using NextSeq 500/550 High Output Kit v2.5 (300 Cycles). The pooled libraries were loaded at 1.5 pM and 1% Phix at 1.5 pM.

### 2.3. Genetic Testing and HRD Assessment

In accordance with European guidelines, all patients underwent *BRCA1/2* genetic testing at the time of the primary diagnosis [7].

Genetic variants were classified based on the ClinVar and Franklin databases or using a custom ad hoc bioinformatic script. Formalin-fixed paraffin-embedded (FFPE) ovarian cancer tissue specimens from patients with a known *BRCA1/2* mutational status were used to assess the HRD status.

The bioinformatic analysis was conducted using a previously validated computational pipeline, as reported by Scaglione et al. [6].

### 2.4. Statistical Analysis

Statistical analyses were performed to identify potential correlations between the HRD status and clinicopathologic features. Descriptive statistics were used to summarize the data, while categorical variables were compared using the Chi-square or Fisher’s exact test. Continuous variables were analyzed using the Student’s *t*-test or Mann–Whitney U test, as appropriate. A *p*-value of <0.05 was considered statistically significant. All calculations were performed using R Statistical Software (v4.2.3; R Core Team 2021).

### 2.5. Age Stratification and Data Sources

Ovarian cancer patients were aggregated from two sources: (1) a real-world cohort collected at our hospital and (2) the publicly available *BRCA1/2* mutated ovarian cancer group obtained from The Cancer Genome Atlas (TCGA) via the Genomic Data Commons (GDC) Data Portal [8].

The HRD status for TCGA samples was derived based on the classification described by Takaya et al. [9].

All patients were grouped into five diagnostic age categories: <40, 40–51, 52–65, 65–70, and 70+ years. Log odds were calculated within each group to assess the association between the diagnostic age and HRD status, and separately for HR-negative (HR-N) and HR-deficient (HR-D) populations.

## 3. Results

### 3.1. Patient Characteristics and Overall HR Score Distribution

Among the fifty-one patients, thirty-nine carried pathogenic *BRCA* variants (twenty-two in *BRCA1* and seventeen in *BRCA2*); four harbored likely pathogenic variants (two in *BRCA1*; two in *BRCA2*), seven had variants of uncertain significance (three in *BRCA1*; four in *BRCA2*), and one was negative for *BRCA* alterations. Variants were annotated according to the reference transcripts NM_007294.4 (*BRCA1*) and NM_000059.4 (*BRCA2*).

A summary of identified *BRCA1/2* variants, including pathogenic, likely pathogenic, and uncertain significance classifications, as reported in ClinVar and Franklin or inferred through bioinformatic prediction tools, is reported in Table 1. The five terms for the pathogenicity of all variants refer to ACMG/AMP recommendations [10].

The baseline demographic and clinical characteristics of the 51 women included in the study cohort are presented in Table 2.

The median age at diagnosis was 56 years (IQR: 51–62). Age was consolidated into three groups (40–50, 51–64, and >65 years) for statistical analysis due to sample size limitations.

Most patients (n = 42, 82%) received treatment with PARP inhibitors. Among them, most were treated with olaparib (n = 35, 83.3%), six were treated exclusively with niraparib, and for one patient enrolled in a clinical trial, we were not informed whether she received olaparib or the placebo.

As previously described [6], we classified patients into three subgroups based on large-scale genomic alterations (LGAs): HR-deficient (HR-D) when LGAs > 20, HR-mild (HR-M) when 15 < LGAs < 19 and HR-negative (HR-N) when LGAs < 14.

Samples identified as negative by our pipeline were labeled as HR-N.

Table 2 summarizes the mutational status of all patients included in the present study. Across the 51 patients analyzed, the HR-D category (score = 2) was the most frequent (47%), followed by the HR-N category (score = 0) (37%) and HR-M category (score = 1) (16%), with no significant association with the *BRCA1/2* gene variant type observed (*p* = 0.72).

Among the 27 patients with a *BRCA1* mutation, the HR-D category was the most prevalent (66.7%), particularly in Class 5 (83.3%), while Class 3 showed an even distribution across categories. All patients carrying a Class 4 alteration belonged to the HR-D category, with no significant association (*p* = 0.51) with the *BRCA1/2* gene variant type.

For the 23 patients with a *BRCA2* mutation, the HR-N category (score = 0) was the most common (52%), particularly in Class 3 (25%), while Class 5 had a more mixed distribution. Patients with non-structural *BRCA* alterations were predominantly classified as the HR-D category (52%), whereas those with structural alterations were more frequently categorized as the HR-N category (67%). The only *BRCA*-negative patient belonged to the HR-N category.

Patients were then categorized into three groups based on the *BRCA1/2* variant status: pathogenic (P), likely pathogenic (LP), and variants of uncertain significance (VUSs). Regarding HRD testing within the *BRCA* mutation cohort, we explored both variant classes and their distributions across the functional domains.

No significant association was observed between the *BRCA1/2* variant classification and HRD positivity.

Among 51 patients with available variant annotations, the most frequently affected functional domains were the DNA-binding domain (DBD; 15 patients, 29%) and exon 11 (10 patients, 20%). Additional variants were in BRCT-related (six patients, 12%), PALB2/RAD51-binding domain (nine patients, 18%), RING (six patients, 12%), and COIL-related regions (two patients, 3.9%). No variants were identified in the isolated PALB2-binding domain. Other less frequent sites included the “other” protein regions (two patients, 3.9%).

To further investigate the biological significance of *BRCA* variants in the context of HRD, we analyzed their distributions across defined functional domains of the *BRCA1* and *BRCA2* genes. The data are shown in Table 3 for the *BRCA1* gene and in Table 4 for the *BRCA2* gene.

### 3.2. Distribution of Mutations in the BRCA1 and BRCA2 Genes

**Figure 1:**
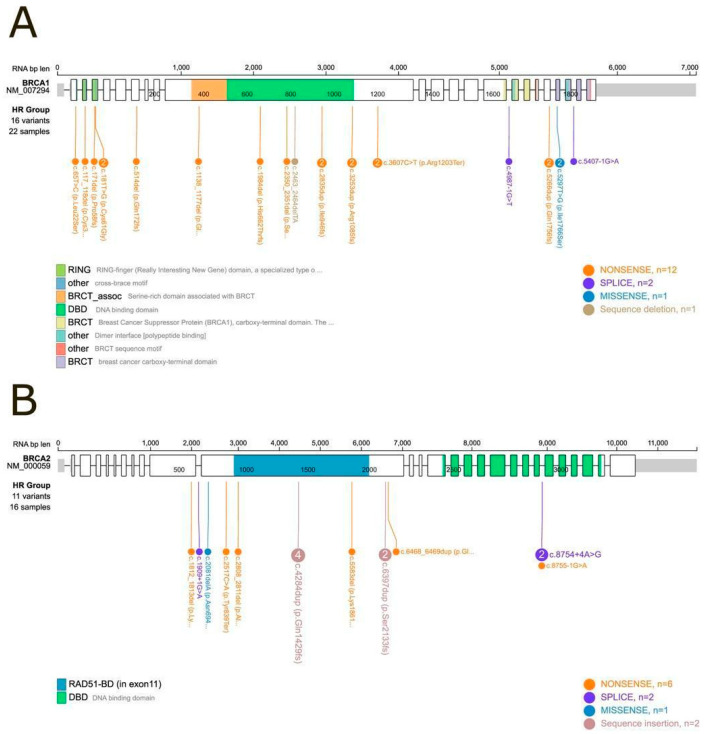
Lollipop plot of *BRCA1* and *BRCA2* mutational domains. (**A**) *BRCA1*: Among the 24 samples with a Class 5 or Class 4 (pathogenic or likely pathogenic) mutation, 22 could be positioned within specific mutational domains. Two samples (IDs 10 and 20) carried large deletions that prevented precise domain mapping. (**B**) *BRCA2*: Among the nineteen samples with a Class 5 or Class 4 mutation, sixteen were successfully mapped to defined protein domains, while three samples (IDs 13, 14, and 52) harbored large deletions that could not be localized within the domain structure.

Among the Class 4 and Class 5 variants, the distribution was as follows (data shown in Figure 1):

• Among the 22 samples harboring *BRCA1* mutations, 16 distinct variants were identified. These included twelve nonsense variants, two splice-site variants, one missense variant, and one deletion.
• Among the 16 samples harboring *BRCA2* mutations, 11 distinct variants were identified. These comprised six nonsense variants, two splice-site variants, one missense variant, and two insertions.

### 3.3. HR Status and BRCA Mutational Domain Correlation

**Figure 2:**
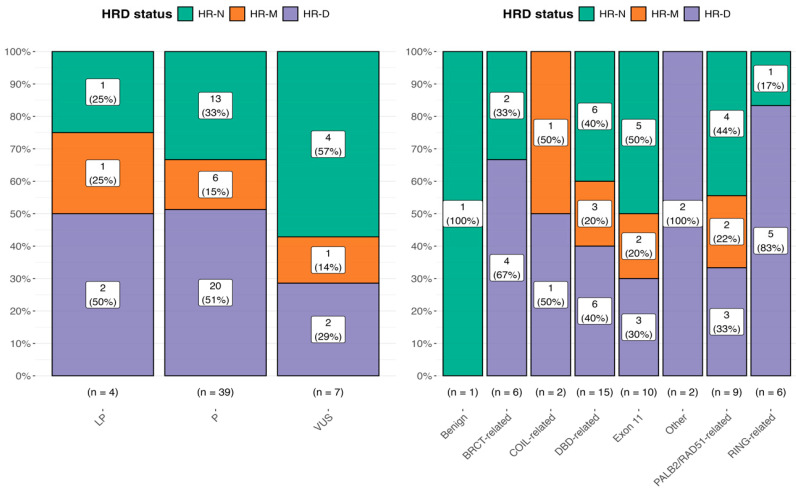
Distribution of the HRD status among ClinVar mutational classes (left panel) and across *BRCA1/2* functional domains (right panel). Percentages within the bars represent the proportion of each mutational class/BRCA domain within the corresponding HRD status group. The right panel considers all 51 HRD tests, including also sample 38 (benign) with an HRD-N status.

We performed a statistical comparison using Chi-square tests for each mutational class to evaluate the association between the HR status and *BRCA* mutational class. The results are shown in Figure 2, where the bar plot displays the distribution of mutational classes (VUSs, LP, and P) across different HR status categories.

Statistical comparisons were performed using Chi-square tests for each mutational class, with the following *p*-values: *p* = 0.368 for VUS, ***p = 0.0231*** for P, and *p* = 0.779 for LP. Effect sizes were computed using Pearson’s method, with estimates of 0.471, 0.402, and 0.333, respectively. For the *BRCA* domain, we found the following result: *p* = 0.14 for the BRCT-related domain, *p* = 0.61 for the COIL-related domain, *p* = 0.55 for the DBD-related domain, *p* = 0.50 for exon 11, *p* = 0.14 for other, *p* = 0.72 for the PALB2/RAD51-related domain, and ***p = 0.03*** for the RING-related domain.

Finally, when we correlated the HR score, categorized as HR-N = 0, HR-M = 1, and HR-D = 2, with the *BRCA* mutation classes, Class 3 variants (VUSs) displayed the highest proportion of HR-N samples. No significant association was found, with further confirmation that HRD positivity does not consistently align with the *BRCA* variant classification.

Moreover, no significant differences were observed between Class 4 and Class 5 variants regarding the HR-D status, which resulted as an HRD in approximately 50% of patients in both the LP and P categories, respectively. Of note, this behavior was also confirmed using the 29 *BRCA1/2* mutated TCGA ovarian cancer samples (data not shown).

As reported in Table 5, no significant association emerged by evaluating the localization of variants in *BRCA* domains with the HR scores.

Finally, we further investigated the concordance between the HRD score and the same type of mutation found in different patients with *BRCA1/2* mutations. The following Table 6 clearly shows that in the presence of the same mutation, the scoring of HRD was not always the same. Arbitrarily, we considered (1) fully concordant samples as those with the same mutation and same HRD score average; (2) concordant samples as those with the same mutation but with a moderate or high HRD score; and (3) discordant samples as those with the same *BRCA1/2* alteration and divergent HRD scores.

As shown, about 50% of patients with the same types of mutations were not in agreement in terms of the HRD score.

### 3.4. Other Biomarkers

**Figure 3:**
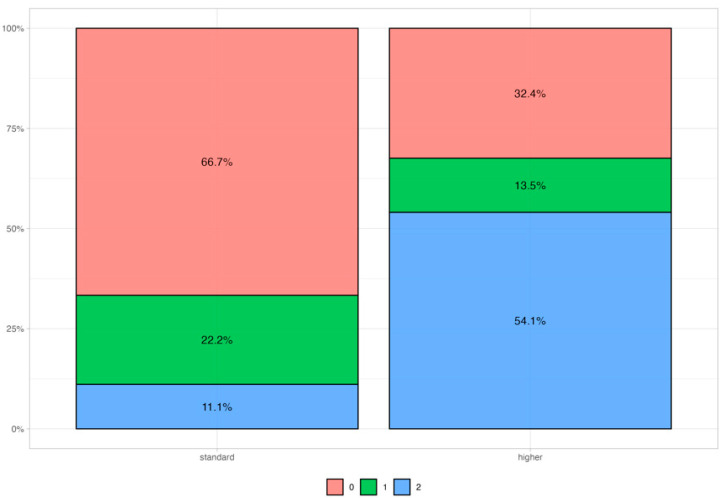
CA15.3 distribution among patients with 3 different HR scores. 0 = HR-N (red); 1 = HR-M (green); 2 = HR-D (blue). Standard values are below 23.4 U/mL.

We also collected information from pre-surgery blood samples for four different biomarkers: CA125 (0.00-24.80 U/mL), CA15.3 (0.00-23.40 U/mL), CA19.9 (up to 35 U/mL), and CEA (0.00–5.0 ng/mL). We found a trend (*p* = 0.083) in the correlation between a high value of CA15.3 and the HR status, as shown in Table 7 and Figure 3.

### 3.5. Diagnostic Age Patterns

The lack of full concordance between the type of mutation found in the different patients and HRD score, led us to investigate which additional factors could influence the HRD pattern of our ovarian cancer patients. Therefore, we analyzed possible correlations between the age of the patients and HRD status.

**Figure 4:**
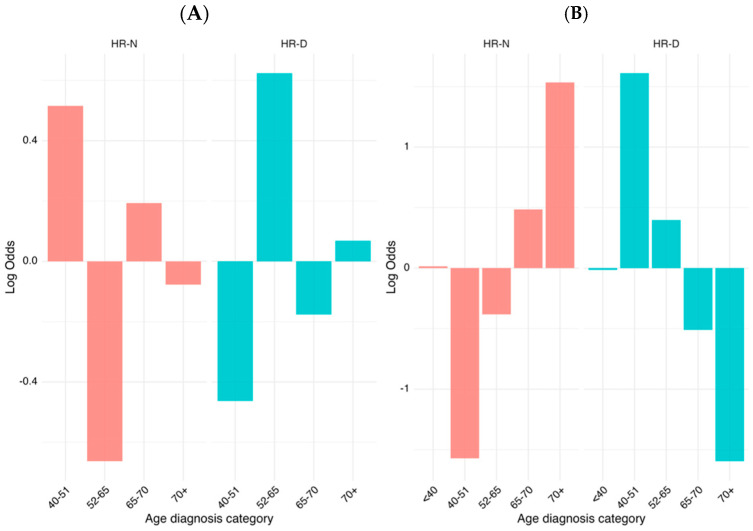
Log odds weight of the HR status across diagnostic age groups of HGSOC patients. (**A**) Log odd ratio distribution of the HR status stratified by the diagnostic age within the current cohort. (**B**) The analysis includes patients from our real-world ovarian cancer cohort combined with publicly available data from patients with *BRCA1/2* mutations from The Cancer Genome Atlas (TCGA). Patients were stratified into five diagnostic age categories (<40, 40–51, 52–65, 65–70, and 70+ years). Log odds values are shown separately for the HR-negative (HR-N, left) and HR-deficient (HR-D, right) subgroups, highlighting contrasting age-associated patterns of HRD prevalence. The right-side chart uses a different scale (−1.5 to 1.5) compared to the left chart (−0.6 to 0.6), indicating different magnitudes of effects being measured.

We conducted a comparative analysis of age at diagnosis categories between two distinct groups, HR-N and HR-D, by utilizing weighted log odds ratios. This statistical method is particularly advantageous as it accounts for both the strength and the variability of associations, providing a more reliable estimate when compared to standard approaches. By incorporating weighted log odds, we can more effectively highlight significant differences in the distribution of age categories between the groups while adjusting for sampling variability. The weighted log odds ratio analysis revealed significant age-related differences in the distribution of HR-N and HR-D group memberships. The 40–51 and 65–70 age categories showed a strong association with HR-N, while the 52–65 category was more enriched in HR-D, with the highest positive log odds values observed across visualizations (Figure 4A). By expanding the sample with data from TCGA cohort, we refined and validated these findings further, ensuring that the identified associations were both statistically robust and biologically meaningful (Figure 4B). As shown, the association between some age categories and HRD score was not completely homogeneous between TCGA and our samples. This behavior can be due to the different sample sizes within the two groups.

This study underscores the importance of considering age as a key factor when investigating the dynamics between the HR-N and HR-D status, and highlights the utility of weighted log odds ratios in drawing more accurate and interpretable conclusions in epidemiological research.

## 4. Discussion

HRD is a key biomarker for predicting the tumor response to PARPis and platinum-based chemotherapy, and it is commonly identified using genomic scars obtained from FFPE samples. These scars reflect past defects in homologous recombination (HR), making them useful indicators of the historical HRD status [11]. HRD is a dynamic status that can change over time. Tumors may acquire resistance through mechanisms like *BRCA* reversion mutations or a restoration of HR function, which genomic scars cannot detect. As a result, relying solely on genomic scars poses a risk of false-positive HRD classifications, leading to suboptimal treatment decisions.

To date, numerous works have been published regarding evaluations of the different methodologies available to define the HRD status [12,13]. However, there is not yet a univocal gold standard assay capable of determining the HRD status, and the major problem concerns the use of commercial tests that use different metrics to categorize patients as HRD or homologous recombination-negative (HR-N) based on a numerical score [6,13,14,15].

Although the different metrics for the HRD assessment show a rate of concordance close to 94% between Myriad myChoice and the alternative assays or in between the alternative assays alone [16], there are some pre-analytical or analytical factors that affect the results of both *BRCA1/2* and HRD testing [4,14].

The main papers published in the HRD setting showed that both the quality and time of FFPE samples can impair the level of analysis, with possible errors or a more than 10% failure rate [17] in the assessment of mutational profile.

Moreover, another potential issue arises by using the *BRCA1/2* mutational status as a part of the algorithms scoring the HRD although the *BRCA1/2* mutational status cannot be always correctly detected. Furthermore, the literature data [3] seem to indicate different magnitudes of response to treatments associated with the specific *BRCA1/2* mutation: therefore, the risk of an erroneous classification of the *BRCA1/2* status could affect the prediction of a patient’s response to treatment [6].

Meanwhile, Andrews et al. [18] have recently investigated the concordance among twenty different assays (both in silico and on clinical samples), showing that the median pairwise positive percent agreement (PPA) for the in silico analysis was 74% and the pairwise negative percent agreement (NPA) was 81% (64–92%). For the clinical assays, indeed, the PPA was 83% and NPA was 80%. A higher positive agreement on the HRD status calls among those with a *BRCA*1 or *BRCA2* mutation was found. This variability underscores the urgent need for standardization to enhance the reliability and clinical applicability of HRD testing in high-grade serous ovarian cancer (HGSOC). Therefore, factors such as the presence of *BRCA1/2* mutations, *CCNE1* amplifications, and assay-specific algorithms can contribute substantially to discrepancies in HRD status reporting.

All these heterogeneous findings underline that the standardization of the inter NGS-based pipelines is crucial because errors in determining the molecular EOC profile status would lead to a different patient treatments and outcomes. This risk is common for various molecular assays used in routine practice [16]. In keeping with some literature data, our group has recently published that the concordance between the genomic and functional HRD (as evaluated by a RAD51 focus assessment), is high only when the genomic HRD score is high (>55); these data were obtained on a peculiar cohort of patients enrolled within the MITO16-MaNGO-OV-2 trial [4]. This would lead us to speculate that the sole *BRCA1/2* mutation cannot completely drive the genomic scar.

To address these challenges and improve patient stratification, we have previously validated a shallow whole-genome sequencing (sWGS) pipeline capable of categorizing patients into three distinct groups based on large-scale genomic alterations (LGAs): HR-deficient, HR-mild, and HR-negative [6]. This innovative approach leverages the detection of LGAs as a surrogate marker for homologous recombination repair pathway dysfunction. By integrating genomic scar metrics with sWGS data, this method offers a refined framework for stratifying patients beyond conventional binary classifications of HRD-positive or HRD-negative [6].

Therefore, the importance of implementing the actual bioinformatic HRD analysis including biological factors like age along with other prediction models incorporating different clinical–biological parameters, such as the g/t*BRCA*m status, mutations in other HRR pathway genes, methylation status, RAD51 foci, response to platinum-based chemotherapy, and CA125 levels, can allow for more accurate and individualized treatment.

This concept was further strengthened by the evidence obtained in the retrospective cohort of fifty-one patients analyzed in this study, a portion of whom carried identical *BRCA* mutations but differed in their HR status. When correlating the *BRCA1/2* mutational status with the HRD phenotype, approximately 50% of samples sharing the same *BRCA1/2* alteration displayed discordant HR statuses. Thus, as recently highlighted by Polajžer et al. [19] and the MITO16-MaNGO-OV-2 trial [4], it is essential to personalize the HRD assessment rather than assuming a homogeneous HRD status in all patients harboring *BRCA* mutations, since the presence of a *BRCA1* or *BRCA2* mutation alone does not necessarily imply a corresponding HRD phenotype, particularly when the genomic HR impairment is not pronounced.

In particular, the HRD status was detected in 29% of VUS carriers. Instead, the site where the mutation falls within functional domains and the related consequences could have more significant roles in determining the HRD status. In fact, in our cohort of patients the domains that were more frequently associated with the HRD status were the BRCT, COIL and RING-related domains. These data are similar to those published by Marchetti et al. [20]. Therefore, there could be other factors influencing the HRD status. In some studies, the frequency of HRD varies with age, and HRD is more common in premenopausal women. Very recently, a research group [21] showed that the genomic features of HRD, PIK3CA mutations with CNAs, and CNAs are enriched in young women with breast cancer. This research group also observed that there was an increasing frequency of HRDetect-positive cancers with decreasing age: 41% in patients < 40 years of age and 47% in patients < 35 years of age compared with 14% in patients 40–45 years of age.

## 5. Conclusions

This study highlights the importance of age as a key factor influencing HR-N and HR-D dynamics and demonstrates the effectiveness of weighted log odds ratios for improving interpretability in epidemiological analyses. Furthermore, our results confirm that the *BRCA1/2* mutation status alone—regardless of the pathogenic classification—does not reliably predict genomic HRD positivity.

## Figures and Tables

**Table 1:** *BRCA1/2* variants according to major databases or bioinformatic predictions. Missing sample IDs belong to patients whose FFPE samples were not eligible for this analysis.

Sample	Type of Mutation	Type of Alteration	Gene	Protein	dbSNP	Class	HRD Report	HRD Score
1	Tumor + Germline	c.5297T>G	*BRCA1*	p.(Ile1766Ser)	rs80357463	**5**	**HR-N**	**0**
2	Tumor + Germline	c.1909+1G>A	*BRCA2*	-	rs587781629	**5**	**HR-D**	**2**
3	Somatic (Germline ND)	c.476-3C>A	*BRCA2*	-	rs371431745	**3**	**HR-D**	**2**
5	Tumor + Germline	c.3607C>T	*BRCA1*	p.(Arg1203Ter)	rs62625308	**5**	**HR-D**	**2**
6	Somatic (Germline ND)	c.2081delA	*BRCA2*	p.(Asn694IlefsTer36)	NR	**4**	**HR-M**	**1**
7	Somatic (Germline ND)	c.2643_2654del	*BRCA2*	p.(Glu881_Ser884)	NR	**3**	**HR-N**	**0**
8	Tumor + Germline	c.5191G>A	*BRCA1*	p.(Glu1731Lys)	rs397507244	**3**	**HR-M**	**1**
9	Somatic	c.4987-1G>T	*BRCA1*	-	rs730881495	**5**	**HR-D**	**2**
10	Somatic	del *BRCA1* gene	*BRCA1*	-	NR	**5**	**HR-N**	**0**
11	Somatic	c.4284dup	*BRCA2*	p.(Gln1429SerfsTer9)	rs80359439	**5**	**HR-N**	**0**
12	Somatic	c.181T>G	*BRCA1*	p.(Cys61Gly)	rs28897672	**5**	**HR-D**	**2**
13	Somatic (Germline ND)	del exons 3–13	*BRCA2*	-	NR	**5**	**HR-N**	**0**
14	Somatic	del exons 2–13	*BRCA2*	-	NR	**5**	**HR-N**	**0**
15	Tumor + Germline	c.6397dup	*BRCA2*	p.(Ser2133fs)	rs431825342	**5**	**HR-N**	**0**
16	Tumor + Germline	c.117_118del	*BRCA1*	p.(Cys39_Asp40delinsTer)	rs80357972	**5**	**HR-D**	**2**
17	Tumor + Germline	c.3253dup	*BRCA1*	p.(Arg1085fs)	rs80357517	**5**	**HR-D**	**2**
18	Tumor + Germline	c.8996T>C	*BRCA2*	p.(Leu2999Pro)	rs876660476	**3**	**HR-N**	**0**
19	Tumor + Germline	c.8754+4A>G	*BRCA2*	-	rs81002893	**5**	**HR-D**	**2**
20	Somatic (Germline ND)	del exons 2–14	*BRCA1*	-	NR	**5**	**HR-N**	**0**
21	Tumor + Germline	c.514del	*BRCA1*	p.(Gln172fs)	rs80357872	**5**	**HR-D**	**2**
22	Tumor + Germline	c.5495T>C	*BRCA1*	p.(Val1832Ala)	rs749290001	**3**	**HR-N**	**0**
25	Tumor + Germline	c.4284dup	*BRCA2*	p.(Gln1429SerfsTer9)	rs80359439	**5**	**HR-N**	**0**
26	Tumor + Germline	c.3607C>T	*BRCA1*	p.(Arg1203Ter)	rs62625308	**5**	**HR-M**	**1**
27	Somatic	c.5583del	*BRCA2*	p.(Lys1861_Val1862insTer)	rs397507790	**5**	**HR-D**	**2**
28	Somatic	c.2517C>A	*BRCA2*	p.(Tyr839Ter)	rs80358516	**5**	**HR-N**	**0**
31	Germline	c.2463_2464delTA	*BRCA1*	p.(Asp821fsTer)	NR	**5**	**HR-N**	**0**
32	Germline	c.2835dup	*BRCA1*	p.(Ile946TyrfsTer6)	rs80357519	**5**	**HR-N**	**0**
33	Germline	c.5266dup	*BRCA1*	p.(Gln1756ProfsTer74)	rs80357906	**5**	**HR-D**	**2**
34	Germline	c.1812_1813del	*BRCA2*	p.(Lys604AsnfsTer11)	NR	**4**	**HR-N**	**0**
35	Tumor + Germline	c.171delG	*BRCA1*	p.(Pro58fs)	rs80357660	**5**	**HR-D**	**2**
37	Germline	c.5297T>G	*BRCA1*	p.(Ile1766Ser)	rs80357463	**5**	**HR-D**	**2**
38	-	**Negative**	*-*	-	-	**-**	**HR-N**	**0**
39	Tumor + Germline	c.4284dupT	*BRCA2*	p.(Gln1429SerfsTer9)	rs80359439	**5**	**HR-M**	**1**
40	Tumor + Germline	c.2808_2811del	*BRCA2*	p.(Ala938ProfsTer21)	rs80359351	**5**	**HR-M**	**1**
41	Tumor + Germline	c.5266dup	*BRCA1*	p.(Gln1756ProfsTer74)	rs80357906	**5**	**HR-D**	**2**
42	Somatic (Germline ND)	c.649del	*BRCA1*	p.(Ser217ValfsTer17)	rs878854963	**3**	**HR-D**	**2**
43	Tumor + Germline	c.181T>G	*BRCA1*	p.(Cys61Gly)	rs28897672	**5**	**HR-D**	**2**
44	Tumor + Germline	c.8754+4A>G	*BRCA2*	-	rs81002893	**5**	**HR-M**	**1**
45	Tumor + Germline	c.9052_9057del	*BRCA2*	p.(Lys3019_Ser3020del)	rs786202063	**3**	**HR-N**	**0**
46	Somatic (Germline ND)	c.5407-1G>A	*BRCA1*	-	rs80358029	**5**	**HR-D**	**2**
47	Somatic	c.1984del	*BRCA1*	p.(His662ThrfsTer39)	NR	**4**	**HR-D**	**2**
48	Tumor + Germline	c.4284dup	*BRCA2*	p.(Gln1429SerfsTer9)	rs80359439	**5**	**HR-D**	**2**
49	Tumor + Germline	c.6468_6469delTC	*BRCA2*	p.(Gln2157fsTer)	rs80359596	**5**	**HR-N**	**0**
50	Somatic	c.8755-1G>A	*BRCA2*	-	rs81002812	**5**	**HR-N**	**0**
51	Somatic	c.1138_1177del	*BRCA1*	p.(Gln380Ter)	NR	**4**	**HR-D**	**2**
52	Somatic (Germline ND)	del exons 14-15	*BRCA2*	-	NR	**5**	**HR-M**	**1**
53	Tumor + Germline	c.6397dup	*BRCA2*	p.(Ser2133fs)	rs431825342	**5**	**HR-D**	**2**
54	Tumor + Germline	c.2835dup	*BRCA1*	p.(Ile946TyrfsTer6)	rs80357519	**5**	**HR-M**	**1**
56	Tumor + Germline	c.3253dup	*BRCA1*	p.(Arg1085fs)	rs80357517	**5**	**HR-D**	**2**
57	Tumor + Germline	c.65T>C	*BRCA1*	p.(Leu22Ser)	rs80357438	**5**	**HR-D**	**2**
58	Tumor + Germline	c.2350_2351del	*BRCA1*	p.(Ser784ValfsTer5)	rs397508960	**5**	**HR-D**	**2**

**NR** = not reported; **HR-D** = deficient; **HR-M** = mild; **HR-N** = negative; **ND** = not performed.

**Table 2:** Patient characteristics.

Characteristic	N = 51 ^1^
**Mutated *BRCA* gene**	
*BRCA1*	27 (54%)
*BRCA2*	23 (46%)
Benign	1
**Mutation type**	
Frameshift del	14 (28%)
Frameshift dup	12 (24%)
LGR	6 (12%)
SNV—missense	8 (16%)
SNV—nonsense	3 (6.0%)
SNV—splice site	7 (14%)
Wild Type	1
**HRD status**	
0 (HR-N)	19 (37%)
1 (HR-M)	8 (16%)
2 (HR-D)	24 (47%)
**Somatic VAF (%)**	79 (63–85)
Not available *	19
**Onset age (years)**	56 (51–62)
**Age classes (years)**	
40–50	11 (22%)
51–64	30 (59%)
>65	10 (20%)
**CA125 (U/mL)**	1376 (497–3410)
Not available	2
**CA15.3 (U/mL)**	47 (29-84)
Not available	3
**CA19.9 (U/mL)**	6 (3–12)
Not available	2
**CEA baseline (ng/mL)**	1.30 (0.80–2.20)
Not available	5
**Mutation class**	
3 (VUS)	7 (14%)
4 (LP)	4 (8.0%)
5 (P)	39 (78%)
Benign	1
**Mutation class (group)**	
>3 (LP or P)	43 (86%)
3 (VUS)	7 (14%)
Benign	1
**Treatment**	
First-line SoC	51 (100%)
**PARPi maintenance (n = 42)**	
Olaparib	35 (83.3%)
Niraparib	6 (14.3%)
Unknown	1 (2.4%)
**Functional domain**	
Benign	1 (2%)
BRCT-related	6 (12%)
COIL-related	2 (3.9%)
DBD-related	15 (29%)
Exon 11	10 (20%)
Other	2 (3.9%)
PALB2/RAD51-related	9 (18%)
RING-related	6 (12%)

^1^ *n* (%); median (Q1, Q3); * the data were not available within clinical report OR CNV alteration.

**Table 3:** Distribution of functional domains in the *BRCA1* gene.

Sample	*BRCA* Domain	Type of Alteration	Class	HRD Report
1	BRCT	c.5297T>G	5	HR-N
37				HR-D
22		c.5495T>C	3	HR-N
33		c.5266dup	5	HR-D
41				HR-D
46		c.5407-1G>A	5	HR-D
8	COIL COIL	c.5191G>A	3	HR-M
9		c.4987-1G>T	5	HR-D
10	RING-DBD-COIL COIL-BRCT	del *BRCA1* gene	5	HR-N
12	RING	c.181T>G	5	HR-D
43				HR-D
16		c.117_118del	5	HR-D
35		c.171delG	5	HR-D
57		c.65T>C	5	HR-D
20	RING-DBD	del exons 2–14	5	HR-N
17	DBD	c.3253dup	5	HR-D
56				HR-D
31		c.2463_2464delTA	5	HR-N
32		c.2835dup	5	HR-N
54				HR-M
47		c.1984del	4	HR-D
51		c.1138_1177del	4	HR-D
58		c.2350_2351del	5	HR-D
5	EXON 11	c.3607C>T	5	HR-D
26				HR-M
21	OTHER	c.514del	5	HR-D
42		c.649del	3	HR-D

**Table 4:** Distribution of functional domains in the *BRCA2* gene.

Sample	*BRCA* Domain	Type of Alteration	Class	HRD Report
18	DBD	c.8996T>C	3	HR-N
19		c.8754+4A>G	5	HR-D
44				HR-M
45		c.9052_9057del	3	HR-N
50		c.8755-1G>A	5	HR-N
52		del exons 14-15	5	HR-M
11	RAD51-BD	c.4284dup	5	HR-N
25				HR-N
39				HR-M
48				HR-D
27		c.5583del	5	HR-D
40		c.2808_2811del	5	HR-M
3	PALB2-BD	c.476-3C>A	3	HR- D
13	PALB2-BD-RAD51-BD	del exons 3–13	5	HR-N
14		del exons 2–13	5	HR-N
2	EXON 11	c.1909+1G>A	5	HR-D
6		c.2081delA	4	HR-M
7		c.2643_2654del	3	HR-N
15		c.6397dup	5	HR-N
53				HR-D
28		c.2517C>A	5	HR-N
34		c.1812_1813del	4	HR-N
49		c.6468_6469delTC	5	HR-N

**Table 5:** Association between the HR status and *BRCA1/2* functional domain.

Characteristic	HR-D N = 24	HR-M N = 8	HR-N N = 19	*p*-Value ^1^
**Domain, n (%)**				0.32
Benign	0 (0)	0 (0)	1 (5.3)	
BRCT2	4 (17)	0 (0)	2 (11)	
COIL COIL	1 (4.2)	1 (13)	0 (0)	
DBD	6 (25)	3 (38)	5 (26)	
Exon 11	3 (13)	2 (25)	5 (26)	
Other	2 (8.3)	0 (0)	0 (0)	
PALB2-BD	1 (4.2)	0 (0)	0 (0)	
PALB2-BD-RAD51-BD	0 (0)	0 (0)	2 (11)	
RAD51-BD	2 (8.3)	2 (25)	2 (11)	
RING	5 (21)	0 (0)	0 (0)	
RING-DBD	0 (0)	0 (0)	1 (5.3)	
RING-DBD-COIL COIL-BRCT	0 (0)	0 (0)	1 (5.3)	
**Domain_group, n (%)**				0.60
Benign	0 (0)	0 (0)	1 (5.3)	
BRCT-related	4 (17)	0 (0)	2 (11)	
COIL-related	1 (4.2)	1 (13)	0 (0)	
DBD-related	6 (25)	3 (38)	6 (32)	
Exon 11	3 (13)	2 (25)	5 (26)	
Other	2 (8.3)	0 (0)	0 (0)	
PALB2/RAD51-related	3 (13)	2 (25)	4 (21)	
RING-related	5 (21)	0 (0)	1 (5.3)	
Mutation class, n (%)				0.75
LP	2 (8.3)	1 (13)	1 (5.6)	
P	20 (83)	6 (75)	13 (72)	
VUS	2 (8.3)	1 (13)	4 (22)	
Unknown	0	0	1	

^1^ Fisher’s exact test.

**Table 6:** Concordance or discordance between specific *BRCA1/2* mutations and the HRD assessment.

Type of Alteration	Gene	Class	*BRCA* Domain	Sample	HRD Report	Level of Concordance
c.5297T>G	*BRCA1*	**5**	BRCT	1	**HR-N**	**Discordant**
				37	**HR-D**	
c.3607C>T	*BRCA1*	**5**		5	**HR-D**	**Concordant**
				26	**HR-M**	
c.4284dup	*BRCA2*	**5**	RAD51-BD	11	**HR-N**	
				25	**HR-N**	**Discordant**
				39	**HR-M**	
				48	**HR-D**	
c.181T>G	*BRCA1*	**5**	RING	12	**HR-D**	**Fully concordant**
				43	**HR-D**	
del exons 3–13	*BRCA2*	**5**	PALB2-BD-RAD51-BD	13	**HR-N**	**Fully concordant**
del exons 2–13	*BRCA2*	**5**	PALB2-BD-RAD51-BD	14	**HR-N**	
c.6397dup	*BRCA2*	**5**		15	**HR-N**	**Discordant**
				53	**HR-D**	
c.3253dup	*BRCA1*	**5**	DBD	17	**HR-D**	**Fully concordant**
				56	**HR-D**	
c.8754+4A>G	*BRCA2*	**5**	DBD	19	**HR-D**	**Concordant**
				44	**HR-M**	
c.2835dup	*BRCA1*	**5**	DBD	32	**HR-N**	**Discordant**
				54	**HR-M**	
c.5266dup	*BRCA1*	**5**	BRCT	33	**HR-D**	**Fully concordant**
				41	**HR-D**	

**Table 7:** HR score and biomarker associations.

Characteristic	HR SCORE			*p*-Value ^2^
	**0 N = 19 ^1^**	**1 N = 8 ^1^**	**2 N = 24 ^1^**	
Age at diagnosis	56 (48, 62)	58 (52, 61)	56 (52, 61)	>0.9
CA125 (U/mL)	818 (308, 2371)	1888 (270, 3523)	1486 (734, 4591)	0.3
Unknown	1	0	1	
CA 15.3 (U/mL)	30 (14, 54)	49 (19, 131)	59 (34, 114)	**0.083**
Unknown	1	1	1	
CA19.9 (U/mL)	5 (2, 24)	8 (6, 10)	6 (1, 11)	0.8
Unknown	1	0	1	
CEA (ng/mL)	1.60 (0.60, 2.15)	0.95 (0.79, 2.30)	1.25 (0.80, 2.20)	0.9
Unknown	3	0	2	

^1^ n (%); median (Q1, Q3), ^2^ Fisher’s exact test; Kruskal–Wallis rank sum test.

## Data Availability

The original contributions presented in this study are included in the article. Further inquiries can be directed to the corresponding author(s).

## References

[1] Capoluongo E., Ellison G., López-Guerrero J.A., Penault-Llorca F., Ligtenberg M.J.L., Banerjee S., Singer C., Friedman E., Markiefka B., Schirmacher P. (2017). Guidance Statement On BRCA1/2 Tumor Testing in Ovarian Cancer Patients. Semin. Oncol..

[2] Buonaiuto R., Neola G., Caltavituro A., Longobardi A., Mangiacotti F.P., Cefaliello A., Lamia M.R., Pepe F., Ventriglia J., Malapelle U. (2024). Efficacy of PARP inhibitors in advanced high-grade serous ovarian cancer according to BRCA domain mutations and mutation type. Front. Oncol..

[3] Labidi-Galy S.I., Rodrigues M., Sandoval J.L., Kurtz J.E., Heitz F., Mosconi A.M., Romero I., Denison U., Nagao S., Vergote I. (2023). Association of location of BRCA1 and BRCA2 mutations with benefit from olaparib and bevacizumab maintenance in high-grade ovarian cancer: Phase III PAOLA-1/ENGOT-ov25 trial subgroup exploratory analysis. Ann. Oncol..

[4] Pellegrino B., Capoluongo E.D., Bagnoli M., Arenare L., Califano D., Scambia G., Cecere S.C., Silini E.M., Scaglione G.L., Spina A. (2025). Unraveling the complexity of HRD assessment in ovarian cancer by combining genomic and functional approaches: Translational analyses of MITO16-MaNGO-OV-2 trial. ESMO Open.

[5] Nunziato M., Scaglione G.L., Di Maggio F., Nardelli C., Capoluongo E., Salvatore F. (2023). The performance of multi-gene panels for breast/ovarian cancer predisposition. Clin. Chim. Acta..

[6] Scaglione G.L., Pignata S., Pettinato A., Paolillo C., Califano D., Scandurra G., Lombardo V., Di Gaudio F., Pecorino B., Mereu L. (2023). Homologous Recombination Deficiency (HRD) Scoring, by Means of Two Different Shallow Whole-Genome Sequencing Pipelines (sWGS), in Ovarian Cancer Patients: A Comparison with Myriad MyChoice Assay. Int. J. Mol. Sci..

[7] Vergote I., González-Martín A., Ray-Coquard I., Harter P., Colombo N., Pujol P., Lorusso D., Mirza M.R., Brasiuniene B., Madry R. (2022). European experts consensus: BRCA/homologous recombination deficiency testing in first-line ovarian cancer. Ann. Oncol..

[8] Cancer Genome Atlas Research Network (2011). Integrated genomic analyses of ovarian carcinoma. Nature.

[9] Takaya H., Nakai H., Takamatsu S., Mandai M., Matsumura N. (2020). Homologous recombination deficiency status-based classification of high-grade serous ovarian carcinoma. Sci. Rep..

[10] Richards S., Aziz N., Bale S., Bick D., Das S., Gastier-Foster K., Grody W.W., Hegde M., Lyon E., Spector E. (2015). Standards and guidelines for the interpretation of sequence variants: A joint consensus recommendation of the American College of Medical Genetics and Genomics and the Association for Molecular Pathology. Genet. Med..

[11] Ledermann J.A., Matias-Guiu X., Amant F., Concin N., Davidson B., Fotopoulou C., González-Martin A., Gourley C., Leary A., Lorusso D. (2024). ESGO-ESMO-ESP consensus conference recommendations on ovarian cancer: Pathology and molecular biology and early, advanced and recurrent disease. Ann. Oncol..

[12] Mangogna A., Munari G., Pepe F., Maffii E., Giampaolino P., Ricci G., Fassan M., Malapelle U., Biffi S. (2023). Homologous Recombination Deficiency in Ovarian Cancer: From the Biological Rationale to Current Diagnostic Approaches. J. Pers. Med..

[13] Quesada S., Penault-Llorca F., Matias-Guiu X., Banerjee S., Barberis M., Coleman R.L., Colombo N., DeFazio A., McNeish I.A., Nogueira-Rodrigues A. (2025). Homologous recombination deficiency in ovarian cancer: Global expert consensus on testing and a comparison of companion diagnostics. Eur. J. Cancer..

[14] Capoluongo E.D., Pellegrino B., Arenare L., Califano D., Scambia G., Beltrame L., Serra V., Scaglione G.L., Spina A., Cecere S.C. (2022). Alternative academic approaches for testing homologous recombination deficiency in ovarian cancer in the MITO16A/MaNGO-OV2 trial. ESMO Open.

[15] Roma C., Abate R.E., Sacco A., Califano D., Arenare L., Bergantino F., Pisano C., Cecere S.C., Scambia G., Lorusso D. (2024). Harmonization of homologous recombination deficiency testing in ovarian cancer: Results from the MITO16A/MaNGO-OV2 trial. Eur. J. Cancer..

[16] Pfarr N., von Schwarzenberg K., Zocholl D., Merkelbach-Bruse S., Siemanowski J., Mayr E.M., Herold S., Kleo K., Heukamp L.C., Willing E.M. (2024). High Concordance of Different Assays in the Determination of Homologous Recombination Deficiency-Associated Genomic Instability in Ovarian Cancer. JCO Precis. Oncol..

[17] Romeya M., Rodepetera F., Hattesohla A., Kaiserb K., Teply-Szymanskia J., Heitzc F., Staeblere A., Serraf V., Grassa A., Marmeg F. (2024). Systematic Analysis of Homologous Recombination Deficiency Testing in Ovarian Cancer-Development of Recommendations for Optimal Assay Performance. Mod. Pathol..

[18] Andrews H.S., McShane L.M., Kohn E.C., Arend R., Karlovich C., Kincaid K., Laird A.D., Li M.C., Sokol E.S., Starks E.R. (2024). Analysis of 20 Independently Performed Assays to Measure Homologous Recombination Deficiency (HRD) in Ovarian Cancer: Findings from the Friends’ HRD Harmonization Project. JCO Oncol. Adv..

[19] Polajžer S., Černe K. (2025). Precision Medicine in High-Grade Serous Ovarian Cancer: Targeted Therapies and the Challenge of Chemoresistance. Int. J. Mol. Sci..

[20] Marchetti C., Fagotti A., Fruscio R., Cassani C., Incorvaia L., Perri M.T., Sassu C.M., Camnasio C.A., Giudice E., Minucci A. (2025). Benefit from maintenance with PARP inhibitor in newly diagnosed ovarian cancer according to BRCA1/2 mutation type and site: A multicenter real-world study. ESMO Open.

[21] Luen S.J., Viale G., Nik-Zainal S., Savas P., Kammler R., Dell’Orto P., Biasi O., Degasperi A., Brown L.C., Láng I. (2023). Genomic characterisation of hormone receptor-positive breast cancer arising in very young women. Ann. Oncol..

